# Supplementary material for: Exploring the Potential of Anthocyanin-Based Edible Coatings in Confectionery—Temperature Stability, pH, and Biocapacity
Source: Foods. 2024 Aug 2;13(15):2450. doi: 10.3390/foods13152450 (PMC11312276; doi:10.3390/foods13152450)
Supplement: Supplementary file 1 [file foods-13-02450-s001.zip › foods-3105830-supplementary.pdf]

foods MDPI

Supplementary Materials

Exploring the Potential of Anthocyanin-Based Edible Coatings in Confectionery – Temperature Stability, pH and Bioactivity

Carmo Serrano [1,2,*,†], Beatriz Lamas[3†], M. Conceição Oliveira[4] and Maria Paula Duarte[3]

[1] Instituto Nacional de Investigação Agrária e Veterinária (INIAV, I.P.), Av. da República, 2780-157 Oeiras, Portugal; carmo.serrano@iniav.pt (C.S.);
[2] LEAF–Linking Landscape: Environment, Agriculture and Food–Research Center, Instituto Superior de Agronomia, Associated Laboratory TERRA, Universidade de Lisboa, Tapada da Ajuda, 1349-017 Lisboa, Portugal;
[3] The Mechanical Engineering and Resource Sustainability Center (MEtRICs), Chemistry Department, NOVA School of Science and Technology, Universidade NOVA de Lisboa, 2829-516 Caparica, Portugal; b.lamas@campus.fct.unl.pt (B.L); mpcd@fct.unl.pt (M.P.D.).
[4] Centro de Química Estrutural, Institute of Molecular Sciences, Instituto Superior Técnico, Universidade de Lisboa, 1049-001 Lisboa, Portugal; conceicao.oliveira@tecnico.ulisboa.pt (M.C. Oliveira)

* Correspondence: carmo.serrano@iniav.pt
† The two authors contributed equally to the article

Table S1. HPLC-DAD-HRMS of acylated anthocyanins identified in the extracts of: purple sweet potato – pulp (PSP) purple sweet potato – peel (PP), black carrot (DC), radish (R), sorghum (S) and dye factory (DF) sample.

t_R (min)	λ_{VIS} (nm)	λ_{UV} (nm)	λ_{Acyl} (nm)	Ionic Formula	[M]$^+$ [*m*/*z* ±Δ ppm; mSigma]	MS/MS [*m*/*z* ±Δ ppm ; attribution]	Probable compound
**PSP and PP extracts**							
6.22	518	266	-	$[C_{33}H_{41}O_{21}]^+$	773.2148 (-1.3; 14.9)	611.1610 (-0.5) $(C_{27}H_{31}O_{16})^+$<br>449.1098 (-4.3) $[C_{21}H_{21}O_{11}]^+$<br>287.0565 (-5.1) $[C_{15}H_{11}O_6]^+$	Cy 3-*O*-soph-5-*O*-glc
6.92	523	268	-	$[C_{34}H_{43}O_{21}]^+$	787.2300 (-2.0; 13.2)	625.1765 (-0.3) $[C_{28}H_{33}O_{16}]^+$<br>463.1259 (-5.3) $[C_{22}H_{23}O_{11}]^+$<br>301.0724 (-5.8) $[C_{16}H_{13}O_6]^+$	Peo 3-*O*-soph-5-*O*-glc
7.90	518	260	320	$[C_{40}H_{45}O_{23}]^+$	893.2359 (-1.8; 6.6)	731.1840 (-3.0) $[C_{34}H_{35}O_{18}]^+$<br>449.1097 (-4.1) $[C_{21}H_{21}O_{11}]^+$<br>287.0566 (-5.5) $[C_{15}H_{11}O_6]^+$	Cy 3-*O*-(*p*-hydroxy benzoyl soph)-5-*O*-glc
8.44	520	278	326	$[C_{41}H_{47}O_{23}]^+$	907.2511 (-0.7; 6.3)	745.1991 (-2.2) $[C_{35}H_{37}O_{18}]^+$<br>463.1250 (-3.3) $[C_{22}H_{23}O_{11}]^+$<br>301.0720 (-4.3) $[C_{16}H_{13}O_6]^+$	Peo 3-*O*-(*p*-hydroxy benzoyl soph)-5-*O*-glc
8.52	520	274	326	$[C_{42}H_{49}O_{24}]^+$	937.2614 (-0.6; 7.2)	775.2107 (-3.5) $[C_{36}H_{39}O_{19}]^+$<br>463.1256 (-4.5) $[C_{22}H_{23}O_{11}]^+$<br>301.0724 (-5.3) $[C_{16}H_{13}O_6]^+$	Peo 3-*O*-(vaniloyl soph)-5-*O*-glc
8.82	524	284	324	$[C_{43}H_{49}O_{24}]^+$	949.2630 (-2.3; 4.8)	787.2104 (-3.0) $[C_{37}H_{39}O_{19}]^+$<br>449.1096 (-3.4) $[C_{21}H_{21}O_{11}]^+$<br>287.0564 (-5.0) $[C_{15}H_{11}O_6]^+$	Cy 3-*O*-(feruloyl soph)--5-*O*-glc
9.30	520	280	327	$[C_{42}H_{47}O_{24}]^+$	935.2451 (-0.1; 6.3)	773.1931 (-0.8) $[C_{36}H_{37}O_{19}]^+$<br>449.1092 (-2.8) $[C_{21}H_{21}O_{11}]^+$<br>287.0562 (-4.0) $[C_{15}H_{11}O_6]^+$	Cy 3-*O*-(caffeoyl soph)--5-O-glc
9.42	520	280	327	$[C_{44}H_{51}O_{24}]^+$	963.2784 (-2.1; 9.3)	801.2249 (-1.6) $[C_{38}H_{41}O_{19}]^+$<br>463.1252 (-3.8) $[C_{22}H_{23}O_{11}]^+$<br>301.0722 (-5.2) $[C_{16}H_{13}O_6]^+$	Peo 3-*O*-(feruloyl soph)-5-*O*-glc
9.59	537	283	320	$[C_{49}H_{51}O_{26}]^+$	1055.2627 (-0.9; 4.3)	893.2161 (-3.0) $[C_{43}H_{41}O_{21}]^+$<br>449.1099 (-4.5) $[C_{21}H_{21}O_{11}]^+$<br>287.0566 (-5.2) $[C_{15}H_{11}O_6]^+$	Cy 3-*O*-(caffeoyl-*p*-hydroxybenzoyl soph)--5-*O*-glc
9.72	528	295	326	$[C_{43}H_{49}O_{24}]^+$	949.2617 (-1.1; 5.9)	787.2096 (-2.0) $[C_{37}H_{39}O_{19}]^+$<br>433.1252 (-3.8) $[C_{22}H_{23}O_{11}]^+$	Peo 3-*O*-(caffeoyl soph)-5-*O*-glc

						301.0722 (-5.2) $[C_{16}H_{13}O_6]^+$	
9.89	528	290	326	$[C_{50}H_{53}O_{26}]^+$	1069.2833 (-1.3; 13.3)	907.2312 (-2.2) $[C_{44}H_{43}O_{21}]^+$<br>463.1252 (-3.8) $[C_{22}H_{23}O_{11}]^+$<br>301.0723 (-5.2) $[C_{16}H_{13}O_6]^+$	Peo 3-*O*-(caffeoyl-*p*-hydroxybenzoyl soph)--5-*O*-glc
9.87	528	284	326	$[C_{51}H_{55}O_{27}]^+$	1099.2937 (-0.6; 5.3)	937.2420 (-2.2) $[C_{45}H_{45}O_{22}]^+$<br>463.1252 (-4.1) $[C_{22}H_{23}O_{11}]^+$<br>301.0724 (-5. 6) $[C_{16}H_{13}O_6]^+$	Peo 3-*O*-(vaniloyl-caffeoyl soph)-5-*O*-glc
10.30	530	284	326	$[C_{53}H_{57}O_{27}]^+$	1125.3096 (-1.2; 9.7)	963.2571 (-1.8) $[C_{47}H_{47}O_{22}]^+$<br>463.1251 (-3.5) $[C_{22}H_{23}O_{11}]^+$<br>301.0722 (-5.0) $[C_{16}H_{13}O_6]^+$	Peo 3-*O*-(caffeoyl-feruloyl soph)-5-*O*-glc
**BC extract**							
7.46	518	236	-	$[C_{32}H_{39}O_{20}]^+$	743.2042 (-1.7; 2.5)	287.0564 (-4.9) $[C_{15}H_{11}O_6]^+$	Cy 3-*O* xylosylglucosyl--gal
7.78	520	234	-	$[C_{26}H_{29}O_{15}]^+$	581.1516 (-2.7; 5.5)	287.0564 (-4.9) $[C_{15}H_{11}O_6]^+$	Cy 3-*O*-xylosyl-gal
8.03	530	286	330	$[C_{43}H_{49}O_{24}]^+$	949.2622 (-1.5; 27.7)	287.0566 (-5.5) $[C_{15}H_{11}O_6]^+$	Cy 3-*O*-xylosyl-(sinapoylglucosyl)-gal
8.20	528	284	328	$[C_{42}H_{47}O_{23}]^+$	919.2521 (-2.0; 18.8)	287.0570 (-5.8) $[C_{15}H_{11}O_6]^+$	Cy 3-*O*-xylosyl-(feruloyl glucosyl)-gal
8.36	530	298	324	$[C_{44}H_{51}O_{24}]^+$	963.2776 (-1.1; 1.2)	301.0724 (-5.8) $[C_{16}H_{13}O_6]^+$	Peo 3-*O*-xylosyl-(sinapoylglucosyl)-gal
8.44	528	296	322	$[C_{41}H_{45}O_{22}]^+$	889.2397 (-1.0; 1.8)	287.0562 (-4.3) $[C_{15}H_{11}O_6]^+$	Cy 3-*O*-xylosyl-(coumaryl glucosyl)-gal
8.56	528	294	330	$[C_{42}H_{47}O_{22}]^+$	903.2562 (-0.9; 3.9)	271.0612 (-3.9) $[C_{15}H_{11}O_5]^+$	Pg 3-*O*-xylosyl (feruloylglucosyl)-gal
8.58	532	282	324	$[C_{43}H_{49}O_{23}]^+$	933.2659 (-1.0; 6.2)	301.0721 (-4.8) $[C_{16}H_{13}O_6]^+$	Peo 3-*O*-xylosyl (feruloylglucosyl)-gal
**R extract**							
6.90	504	262	-	$[C_{33}H_{41}O_{20}]^+$	757.2193 (-0.9; 15.6)	595.1678 (-3.4) $(C_{27}H_{24}O_{15})^+$<br>433.1154 (-5.8) $[C_{21}H_{21}O_{10}]^+$<br>271.0513 (-3.4) $[C_{15}H_{11}O_5]^+$	Pg 3-*O*-soph-5-glc
9.35	510	286	316	$[C_{45}H_{49}O_{26}]^+$	1005.2501 (-0.6; 3.1)	757.1986 (-1.5) $[C_{36}H_{37}O_{18}]^+$<br>519.1148 (-2.8) $[C_{24}H_{23}O_{13}]^+$<br>271.0612 (-4.2) $[C_{15}H_{11}O_5]^+$	Pg 3-*O*-(caffeoyl-soph)--5-*O*-(malonyl)-glc
9.64	508	280	326	$[C_{42}H_{47}O_{23}]^+$	919.2510 (-0.8; 8.3)	757.1983; -1.2 $[C_{36}H_{37}O_{18}]+$<br>433.1143 (-3.2)$[C_{21}H_{21}O_{10}]+$<br>271.0612; -3.8$[C_{15}H_{11}O_5]+$	Pg 3-*O*-(caffeoyl-soph)--5-*O*-glc
9.96	510	290	318	$[C_{45}H_{49}O_{25}]^+$	989.2562 (-0.5; 10.3)	741.2035 (-1.4) $[C_{36}H_{37}O_{17}]^+$<br>519.1144 (-2.1) $[C_{24}H_{23}O_{13}]^+$<br>271.0611 (-3.9) $[C_{15}H_{11}O_5]^+$	Pg 3-*O*-(*p*-coumaroyl-soph)-5-*O*-(malonyl)--glc
10.04	510	290	324	$[C_{46}H_{51}O_{26}]^+$	1019.2667 (-0.4; 5.7)	771.2143 (-1.6) $[C_{37}H_{39}O_{18}]^+$<br>519.1146 (-2.5) $[C_{24}H_{23}O_{13}]^+$<br>271.0612 (-4.2) $[C_{15}H_{11}O_5]^+$	Pg 3-*O*-(feruloyl soph)--5-*O*-malonyl-glc
10.09	510	290	318	$[C_{45}H_{49}O_{26}]^+$	1005.2501 (-0.6; 3.1)	757.1986 (-1.5) $[C_{36}H_{37}O_{18}]^+$<br>519.1148 (-2.8) $[C_{24}H_{23}O_{13}]^+$<br>271.0612 (-4.2) $[C_{15}H_{11}O_5]^+$	Pg 3-*O*-(caffeoyl-soph)--5-*O*-(malonyl)-glc
10.17	508	272	316	$[C_{42}H_{47}O_{22}]^+$	903.2553 (-0.1; 9.1)	741.2035 (-1.4) $[C_{36}H_{37}O_{17}]^+$<br>433.1142 (-2.1) $[C_{21}H_{21}O_{10}]^+$<br>271.0611 (-3.9) $[C_{15}H_{11}O_5]^+$	Pg 3-*O*-(*p*-coumaroyl) soph-5-*O*-glc
10.21	508	286	320	$[C_{43}H_{49}O_{23}]^+$	933.2660 (-0.1; 2.4)	771.2151 (-1.4) $[C_{37}H_{39}O_{18}]^+$<br>433.1145 (-3.6) $[C_{21}H_{21}O_{10}]^+$<br>271.0612 (-4.0) $[C_{15}H_{11}O_5]^+$	Pg 3-*O*-(feruloyl-soph)--5-*O*-glc
10.58	510	292	318	$[C_{45}H_{49}O_{25}]^+$	989.2562 (-0.5; 10.3)	741.2035 (-1.4) $[C_{36}H_{37}O_{17}]^+$<br>519.1144 (-2.1) $[C_{24}H_{23}O_{13}]^+$<br>271.0611 (-3.9) $[C_{15}H_{11}O_5]^+$	Pg 3-*O*-(*p*-coumaroyl-soph)-5-*O*-(malonyl)- -glc
10.84	510	286	322	$[C_{46}H_{51}O_{26}]^+$	1019.2667 (-0.4; 5.7)	771.2143 (-1.6) $[C_{37}H_{39}O_{18}]^+$<br>519.1146 (-2.5) $[C_{24}H_{23}O_{13}]^+$<br>271.0612 (-4.2) $[C_{15}H_{11}O_5]^+$	Pg 3-*O*-(feruloyl soph)- -5-*O*-(malonyl)-glc
11.15	514	290	318	$[C_{52}H_{55}O_{25}]^+$	1079.3035 (-0.8; 18.5)	917.2501(-0.2) $[C_{46}H_{45}O_{20}]^+$<br>433.1138 (-2.1) $[C_{21}H_{21}O_{10}]^+$<br>271.0612 (-4.1) $[C_{15}H_{11}O_5]^+$	Pg 3-*O*-(*p*-coumaroyl -feruloyl-soph)-5-*O*-glc
11.19	514	286	324	$[C_{53}H_{57}O_{26}]^+$	1109.3135 (-0.2; 17.6)	947.2596 (-0.2) $[C_{47}H_{47}O_{21}]^+$<br>433.1140 (-2.5) $[C_{21}H_{21}O_{10}]^+$<br>271.0608 (-2.8) $[C_{15}H_{11}O_5]^+$	Pg 3-*O*-(diferuloyl soph)-5-*O*-glc
11.32	514	286	324	$[C_{55}H_{57}O_{29}]^+$	1181.2992 (-0.2; 17.6)	933.2460 (-1.3) $[C_{46}H_{45}O_{21}]^+$<br>519.1146; -2.5 $[C_{24}H_{23}O_{13}]^+$<br>271.0612 (-4.0) $[C_{15}H_{11}O_5]^+$	Pg 3-*O*-(feruloyl-caffeoyl -soph) 5-*O*--(malonyl)-glc

11.61	514	286	320	$[C_{55}H_{57}O_{28}]^+$	1165.3035 (-0.4; 15.9)	917.2516 (-1.9) $[C_{46}H_{45}O_{20}]^+$<br>519.1146; -2.5 $[C_{24}H_{23}O_{13}]^+$<br>271.0611; -3.6 $[C_{15}H_{11}O_5]^+$	Pg 3-*O*-(feruloyl-*p*-coumaroyl soph)- 5-*O*---(malonyl)-glc
11.64	514	286	320	$[C_{56}H_{59}O_{29}]^+$	1195.3131 (-0.4; 13.7)	947.2629 (-2.6) $[C_{47}H_{47}O_{21}]^+$<br>519.1146; -2,5 $[C_{24}H_{23}O_{13}]^+$<br>271.0611; -3.6 $[C_{15}H_{11}O_5]^+$	Pg 3-O-(diferuloyl soph)-5-O-(malonyl)- -glc
11.69	514	286	320	$[C_{54}H_{55}O_{27}]^+$	1135.2927 (-0.2; 7.6)	887.2414 (-2.3) $[C_{45}H_{43}O_{19}]^+$<br>519.1148; -2.8 $[C_{24}H_{23}O_{13}]^+$<br>271.0614; -4.6 $[C_{15}H_{11}O_5]^+$	Pg 3-O-(di-coumaroyl soph)-5-O-(malonyl)- -glc
**S extract**							
9.80	472	276	-	$[C_{15}H_{11}O_4]^+$	255.0660 (-3.2; 6.9)	227.0709 (-2.9) $[C_{14}H_{11}O_3]^+$<br>213.0552 (-3.8) $[C_{13}H_9O_3]^+$<br>197.0597 (+0.1) $[C_{13}H_9O_2]^+$<br>171.0452 (-5.5) $[C_{11}H_7O_2]^+$<br>157.0659 (-6.7) $[C_{11}H_9O]^+$<br>121.0279 (-7.8) $[^{0.2}\mathbf{B}^+]$ $[C_7H_5O_2]^+$	Apigeninidin
10.12	474	276	-	$[C_{16}H_{13}O_5]^+$	285.0757 (-0.3; 8.7)	270.0525 (-0.7) $[C_{15}H_{10}O_5]^{+\bullet}$<br>253.0495 (-0.1) $[C_{15}H_9O_4]^+$<br>239.0707 (-1.9) $[C_{15}H_{11}O_3]^+$<br>137.0610 (-8.9) $[C_8H_9O_2]^+$<br>121.0286 (-9.5) $[^{0.2}\mathbf{B}^+]$ $[C_7H_5O_2]^+$	5-*O*-methoxy-luteolinidin
10.17	472	278	-	$[C_{16}H_{13}O_4]^+$	269.0813 (-1.9; 3.2)	254.0580 (-2.4) $[C_{15}H_{10}O_4]^+$<br>226.0617 (-3.3) $[C_{14}H_{10}O_3]^+$<br>197.0608 (-5.6) $[C_{13}H_9O_2]^+$_<br>169.0661 (-6.9) $[C_{12}H_9O_1]^+$<br>144.0574 (-5.4) $(C_{10}H_9O_1]^+$<br>121.0276 (-7.5) $[^{0.2}\mathbf{B}^+]$ $[C_7H_5O_2]^+$	5-*O*-methoxy-apigeninidin
10.49	472	276	-	$[C_{16}H_{13}O_4]^+$	269.0809 (-0.4; 8.4)	254.0577 (-1.3) $[C_{15}H_{10}O_4]^+$<br>226.0629 (-1.8) $[C_{14}H_{10}O_3]^+$<br>197.0608 (-5.5) $[C_{13}H_9O_2]^+$_<br>158.0732 (-3.6) $[C_{11}H_{10}O_1]^+$<br>144.0574 (-5.4) $(C_{10}H_9O_1]^+$<br>121.0292 (-9.5) $[^{0.2}\mathbf{B}^+]$ $[C_7H_5O_2]^+$	7-*O*-methoxy apigeninidin
**DF extract**							
8.44	528	286	324	$[C_{41}H_{47}O_{23}]^+$	907.2511 (-0.7; 6.3)	745.1991 (-2.2) $[C_{35}H_{37}O_{18}]^+$<br>463.1250 (-3.3) $[C_{22}H_{23}O_{11}]^+$<br>301.0720 (-4.3) $[C_{16}H_{13}O_6]^+$	Peo 3-*O*-(*p*-hydroxy benzoyl soph)-5-*O*-glc
8.82	528	284	326	$[C_{43}H_{49}O_{24}]^+$	949.2630 (-2.3; 4.8)	787.2104 (-3.0) $[C_{37}H_{39}O_{19}]^+$<br>449.1096 (-3.4) $[C_{21}H_{21}O_{11}]^+$<br>287.0564 (-5.0) $[C_{15}H_{11}O_6]^+$	Cy 3-*O*-(feruloyl soph)--5-O-glc
8.86	528	286	324	$[C_{42}H_{47}O_{23}]^+$	919.2500 (-0.3; 10.0)	757.1987 (-1.7) $[C_{36}H_{37}O_{18}]^+$<br>449.1091 (-2.8) $[C_{21}H_{21}O_{11}]^+$<br>287.0562 (-4.1) $[C_{15}H_{11}O_6]^+$	Cy 3-*O*-(*p*-coumaroyl soph)-5-*O*-glc
9.30	520	280	326	$[C_{42}H_{47}O_{24}]^+$	935.2451 (-0.1; 6.3)	773.1931 (-0.8) $[C_{36}H_{37}O_{19}]^+$<br>449.1092 (-2.8) $[C_{21}H_{21}O_{11}]^+$<br>287.0562 (-4.0) $[C_{15}H_{11}O_6]^+$	Cy 3-*O*-(caffeoyl soph)--5-O-glc
9.59	528	284	320	$[C_{49}H_{51}O_{26}]^+$	1055.2627 (-0.9; 4.3)	893.2161 (-3.0) $[C_{43}H_{41}O_{21}]^+$<br>449.1099 (-4.5) $[C_{21}H_{21}O_{11}]^+$<br>287.0566 (-5.2) $[C_{15}H_{11}O_6]^+$	Cy 3-*O*-(caffeoyl-*p*-hydroxybenzoyl soph)-5-*O*-glc
9.72	528	295	326	$[C_{43}H_{49}O_{24}]^+$	949.2617 (-1.1; 5.9)	787.2096 (-2.0) $[C_{37}H_{39}O_{19}]^+$<br>433.1252 (-3.8) $[C_{22}H_{23}O_{11}]^+$<br>301.0722 (-5.2) $[C_{16}H_{13}O_6]^+$	Peo 3-*O*-(caffeoyl soph)-5-*O*-glc
9.75	528	286	326	$[C_{42}H_{47}O_{23}]^+$	919.2505 (-0.3; 8.9)	757.1987 (-1.7) $[C_{36}H_{37}O_{18}]^+$<br>449.1091 (-1.9) $[C_{21}H_{21}O_{10}]^+$<br>271.0610 (-3.4) $[C_{15}H_{11}O_5]^+$	Cy 3-*O*-(*p*-coumaroyl soph)-5-*O*-glc
9.84	528	286	328	$[C_{52}H_{55}O_{27}]^+$	1111.2926 (-0.1; 17.3)	949.2400 (-0.3) $[C_{46}H_{45}O_{22}]^+$<br>449.1099 (-4.5) $[C_{21}H_{21}O_{11}]^+$<br>287.0566 (-5.2) $[C_{15}H_{11}O_6]^+$	Cy 3-*O*-(caffeoyl-feruloy soph)-5-*O*-glc
9.91	528	290	327	$[C_{50}H_{53}O_{26}]^+$	1069.2833 (-1.3; 13.3)	907.2312 (-2.2) $[C_{44}H_{43}O_{21}]^+$<br>463.1252 (-3.8) $[C_{22}H_{23}O_{11}]^+$<br>301.0723 (-5.2) $[C_{16}H_{13}O_6]^+$	Peo 3-*O*-(caffeoyl-*p*-hydroxybenzoyl soph)--5-*O*-glc
9.86	528	290	328	$[C_{52}H_{55}O_{27}]^+$	1111.2926 (-0.1; 17.3)	949.2400 (-0.3) $[C_{46}H_{45}O_{22}]^+$<br>463.1245 (-2.1) $[C_{22}H_{23}O_{11}]^+$<br>301.0718 (-3.6) $[C_{16}H_{13}O_6]^+$	Peo 3-*O*-(dicaffeoyl soph)-5-*O*-glc
10.02	528	288	326	$[C_{51}H_{53}O_{26}]^+$	1081.2817 (-0.2; 3.5)	919.2300 (-0.9) $[C_{45}H_{43}O_{21}]^+$<br>449.1092 (-3.0) $[C_{21}H_{21}O_{11}]^+$<br>287.0561 (-3.8) $[C_{15}H_{11}O_6]^+$	Cy 3-*O*-(*p*-coumaroyl-caffeoyl soph)-5-*O*-glc

10.10	528	290	330	$[C_{51}H_{53}O_{26}]^+$	1081.2817 (-0.2; 3.5)	919.2300 (-0.9) $[C_{45}H_{43}O_{21}]^+$<br>433.1092 (-3.0) $[C_{21}H_{21}O_{11}]^+$<br>271.0610 (-3.4) $[C_{15}H_{11}O_5]^+$	Cy 3-*O*-(dicaffeoyl soph)--5-*O*-glc
10.27	528	292	328	$[C_{53}H_{57}O_{27}]^+$	1125.3096 (-1.2; 9.7)	963.2571 (-1.8) $[C_{47}H_{47}O_{22}]^+$<br>463.1251 (-3.5) $[C_{22}H_{23}O_{11}]^+$<br>301.0722 (-5.0) $[C_{16}H_{13}O_6]^+$	Peo 3-O-(caffeoyl-feruloyl soph)-5-*O*-glc
10.46	530	290	330	$[C_{55}H_{57}O_{30}]^+$	1197.2936 (-0.5; 10.3)	949.2410 (-1.2) $[C_{46}H_{45}O_{22}]^+$<br>535.1096 (-2.6) $[C_{24}H_{23}O_{14}]^+$<br>287.0563 (-4.3) $[C_{15}H_{11}O_6]^+$	Cy 3-*O*-(ferulyl-sinapyl soph)-5-*O*-(malonyl glc)
10.62	528	286	328	$[C_{44}H_{51}O_{24}]^+$	963.2784 (-2.1; 9.3)	801.2240 (-0.2) $[C_{38}H_{41}O_{19}]^+$<br>463.1242 (-1.6) $[C_{22}H_{23}O_{11}]^+$<br>301.0720 (-4. 3) $[C_{16}H_{13}O_6]^+$	Peo 3-*O*-(feruloyl soph)-5-*O*-glc
10.91	530	290	328	$[C_{55}H_{57}O_{30}]^+$	1197.2946 (-1.4; 12.3)	949.2415 (-1.9) $[C_{46}H_{45}O_{22}]^+$<br>535.1095 (-2.6) $[C_{24}H_{23}O_{14}]^+$<br>287.0563 (-4.3) $[C_{15}H_{11}O_6]^+$	Cy 3-*O*-(caffeoyl-sinapyl soph)-5-*O*-(malonyl glc)
11.15	530	292	330	$[C_{56}H_{59}O_{30}]^+$	1211.3090 (-0.4; 12.3)	963.2570 (-1.8) $[C_{47}H_{47}O_{22}]^+$<br>535.1096 (-2.6) $[C_{24}H_{23}O_{14}]^+$<br>287.0564 (-4.7) $[C_{15}H_{11}O_6]^+$	Cy 3-O-(feruloyl-sinapyl soph)-5-O-(malonyl glc)
11.24	530	290	326	$[C_{55}H_{57}O_{29}]^+$	1181.2978 (-0.2; 15.3)	933.2472 (-2.6) $[C_{46}H_{45}O_{21}]^+$<br>535.1099 (-2.6) $[C_{24}H_{23}O_{14}]^+$<br>287.0564 (-4.7) $[C_{15}H_{11}O_6]^+$	Cy 3-*O*-(*p*-coumaroyl-sinapyl soph)-5-*O*-(malonyl]-glc

*Cy, cyanidin; Peo, peonidin; Pl, pelargonidin; soph, sophoroside; glc, glucoside; gal, galactoside.
